# A novel multi-stage attack dataset for smart home intrusion detection

**DOI:** 10.1016/j.dib.2026.112770

**Published:** 2026-04-12

**Authors:** Vipin Das, Binoy B Nair

**Affiliations:** a Department of Electronics and Communication Engineering, Amrita School of Engineering, Coimbatore, Amrita Vishwa Vidyapeetham, India; b Amrita School of Artificial Intelligence, Coimbatore, Amrita Vishwa Vidyapeetham, India

**Keywords:** Smart home, Intrusion detection, Network flow, Machine learning

## Abstract

Modern homes are increasingly relying on networks of internet-connected devices for smart home applications. Unlike an enterprise network, such smart home networks typically tend to have lax cybersecurity protocols in place and are prone to cyber-attacks. Attack patterns have also evolved over time, with multi-stage attacks being more difficult to detect and mitigate.

This article presents a novel dataset captured under normal operation as well as from seven different multi-stage attack scenarios that can arise in a smart home environment that hosts end devices of heterogeneous nature. Unlike conventional network flow datasets which describe individual single-stage attacks on devices, this dataset aims to provide researchers with valuable insights into the behaviour of network parameters when each attack scenario includes a combination of attacks. This dataset with a total of 178,831 samples, is split into two parts: Training- consisting of 148,959 samples and Testing- consisting of 29,872 samples. The captured dataset can be used to develop intrusion detection systems capable of detecting and classifying such interleaved attack patterns that include multiple sequential attack steps in a smart home environment. The dataset captures the common network flow parameters in the network and hence, the dataset can be used to extend the research to other type of networks as well.

Specifications Table

**Table:** 

Subject	Computer Sciences
Specific subject area	Smart home, Intrusion detection and classification, Network flow analysis, Multistage attack detection
Type of data	Filtered, Processed
Data collection	The data was collected from a smart home network testbed with a variety of end devices while under normal operation as well as when subjected to different types of cyber-attacks. The network packets logged were processed and converted to tabular form using a custom script.
Data source location	Amrita Vishwa Vidyapeetham, Coimbatore,India
Data accessibility	Repository name: Mendeley Data Data identification number: DOI: 10.17632/x95b37z2vy.1Direct URL to data: https://data.mendeley.com/datasets/x95b37z2vy/1 Data is in comma separated value files with extension .csv and can be directly downloaded and viewed/used without any further processing.
Related research article	None

## 1 Value of the Data

• The dataset presented in the article corresponds to benign and malicious traffic captured from a smart home network. Malicious activities captured in the dataset correspond to multiple multi-stage attack scenarios that are possible in a network of smart home devices.
• Attack scenarios in the dataset are generated using an adversary emulation platform to maintain consistency and correspond to various strategies defined in the MITRE ATT&CK matrix. The attack scenarios reflect the multiple-stage attacks employed by malicious entities in a smart home network.
• The dataset provides valuable insights into how multistage attacks can be carried out in a home network. Inclusion of multiple attack vectors in each scenario provides an opportunity to analyze the network traffic patterns at each attack stage.
• In the presented dataset, each attack scenario, in line with typical multi-stage attacks, employs multiple attacks in a sequence to complete a malicious task. Some of the individual attacks considered in this work are a part of multiple attack scenarios but occur at different steps in the attack sequences based on the attack scenario, allowing for research into detection and identification of multi-stage attacks. This enhances the novelty of research tasks that can be performed using the dataset.
• A machine learning based attack detection workflow that can be used ith the proposed dataset is also presented. The baseline results for a Random Forest based attack detection system are also included to demonstrate the fact that the multi-stage attacks are indeed detectable using the provided features.

## 2 Background

Network traffic data analysis is an important aspect of cybersecurity. As end devices and network capabilities continue to evolve at a rapid pace, cyber threats have also become increasingly complex. The integration of IoT and other ubiquitous 'smart' devices in modern home networks has resulted in such smart homes becoming targets of malicious entities. Design of proper detection and defense mechanisms to secure such smart home networks require details about the network parameters and the changes in the data patterns for the various security events in the network. Modern artificial intelligence based systems are able to detect [1,2] and prevent the advancements of the threat actors when provided with proper training datasets. Datasets such as UNSW-NB15 [3]and other network flow datasets [4] have been widely utilized to develop systems that could detect and classify threats using the network flow information. These widely used datasets incorporate individual single stage attack classes. Various researchers have adopted the datasets to analyze their methodologies in various types of networks. The intrusion detection system presented in [5]highlighted the relevance of using the dataset for detecting attack classes in an IoT network even though the dataset was not completely representative of the characteristics of an IoT device network. Modern datasets such as CICIoT23 [6] dataset utilizes data captured from a network of IoT devices enabling researchers to identify the threats in an IoT network. The UNSW HomeNet [7] dataset captures the features of the various devices in a home network to classify the devices in the network based on the network flow pattern. The datasets currently used by researchers to strengthen the security of a smart home network are either general in nature or are made up of singular type of devices which does not realistically model a modern day smart home. The experimental attacks performed on these devices are limited to single stage attacks and are captured individually for training and testing. However, modern attackers deploy multiple strategies and multi-stage attacks to carry out malicious activities in the target home network necessitating the need for new datasets to develop effective smart home threat detection models.

## 3 Data Description

The dataset was captured from a smart home network testbed with multiple categories of devices such as end devices, attack initiator, attack targets, and a data logger which are connected to a Wi-Fi router [8]. Devices with varying usage pattern and software systems were selected so as to ensure that the attack flows are embedded within a large amount of diverse traffic. The data was captured using Aircrack-ng [9] tool which offers diverse mechanisms to capture and log the flows in a network. A total of approximately three million PCAP files were captured during the entire experiment process. The PCAP data was further filtered using Wireshark [10]. The filter logic was based on the attack scenarios so as to extract features pertaining to the flow among the attacker and the targets. The filtered PCAP files were converted to a tabular format using a custom Python script designed to order the packets based on the IP address and port number information. Each flow is maintained by a 5-tuple dictionary containing the source ip address, destination ip address, source port and ip protocol version. Flows are aggregated as unidirectional tuples to maintain the security related traffic characteristics in transmission. Packets are assigned to a flow based on a strict match of the tuple, ensuring packet aggregation. Flows persist for the entire duration of the packet capture as adversarial traffic may adopt variable timing. This lightweight scheme suits a smart home environment and ensures that the features are derived from well-defined and consistent data.

The script extracted various data level, flag level, and statistical information from the filtered PCAP files. We have discarded the IP addresses and port number from the tabular dataset as they can lead to potential data leak as far as the attack scenario classes are concerned. The features like ip_id and tcp_seq are retained as they could indicate possible strategies in attack scenarios to fragment the packets to evade detection and possibilities of injecting data packets. These features could reveal attempts to fragment malicious payloads and inject out of order data. The dataset contains 23 features among which one is categorical and the rest are all numerical.

The class wise sample count in the training data is given in Table 1. Our experiments were focused on generating two independent training and testing data using the same adversarial emulation methodology and hardware test bed.

**Table 1:** Count of classes in training data.

Class Type	Count
Attack1	2666
Attack2	78,703
Attack3	1972
Attack4	1901
Attack5	2962
Attack6	2322
Attack7	1085
Normal	57,348

The methodology and adversarial emulation were the same among the attack scenarios in the training and testing experiment phases. The attack scenario two experiment during the training phase was conducted using files of large and small sizes while the test phase used a single file of small size. The testing set as a result contained a small number of flows as compared to that of the training set. The count of classes of the data in testing set is given in Table 2. The researchers using the data may use weighted evaluation metrics to interpret the model performance. The use of sampling strategies may also be explored in the model development phase to reduce the influence of the dominant classes in the data. The observed variation in the class counts is a result of independent capture experiments for the training and testing data rather than using random sampling. The normal traffic in the training and the testing data was captured from the internet traffic and other network interactions made by the nodes in the smart home network.

**Table 2:** Count of classes in testing data.

Class Type	Count
Attack1	1525
Attack2	1667
Attack3	1040
Attack4	1012
Attack5	4827
Attack6	1388
Attack7	1231
Normal	17,182

The features represent various parameters of the network flow. Each attack scenario in the experiment is captured in multiple iterations starting with single target machine and ending with attacking all the targets simultaneously. The feature statistics corresponding to the various attack scenarios and normal traffic are listed in Table 6–Table 13. The features from the network packets were extracted accordingly and saved as separate files for any required analysis during the various machine learning tasks. The features in the dataset along with their data types and description are summarized in Table 3.

**Table 3:** Feature types and description.

Feature Name	Type	Remarks
flow_byte_count	Numerical	Byte count in the flow
flow_inter_packet_delay	Numerical	Delay between subsequent packets
flow_mean_packet_size	Numerical	Mean of the packet size in the flow
flow_packet_count	Numerical	Count of the packets in the flow
flow_packet_size_kurtosis	Numerical	Kurtosis of the flow packet size
flow_packet_size_skewness	Numerical	Skewness of the flow packet size
flow_packet_size_stddev	Numerical	Standard deviation of the flow packet size
flow_payload_size_per_packet	Numerical	Size of each packet in the flow
flow_relative_time	Numerical	Time interval between flows
ip_frag	Numerical	Fragmentation status of IP Packet
ip_id	Numerical	IP identifier
ip_protocol	Numerical	IP protocol version
ip_ttl	Numerical	Time to live value of the packet
packet_length	Numerical	Length of the packet
tcp_ack	Numerical	Acknowledgment number
tcp_flag_ack	Numerical/Logical	Flag representing acknowledgment number message
tcp_flag_fin	Numerical/Logical	Flag representing the final packet
tcp_flag_rst	Numerical/Logical	Flag representing reset message
tcp_flag_syn	Numerical/Logical	Synchronization message
tcp_payload_size	Numerical	Size of the TCP Packet
tcp_seq	Numerical	Sequence number of the packet
tcp_window_size	Numerical	TCP window size
tcp_flags	Categorical	Flags representing the connection status as PUSH,URG

The packet flow captures from the network were converted to tabular form using a custom Python script to extract the relevant features. The script utilized a combination of source and destination identifiers to associate communication between two nodes into unique flows. A sample of data extracted from attack traffic is given in Table 4.

**Table 4:** Sample network flow records from one of the attack scenario.

Feature Name	Sample feature values				
packet_length	66	66	66	66	124
ip_protocol	6	6	6	6	6
ip_ttl	64	64	64	64	64
ip_fragment	0	0	0	0	0
ip_id	34527	20432	20432	34529	34540
tcp_flag_syn	0	0	0	0	0
tcp_flag_ack	1	1	1	1	1
tcp_flag_rst	0	0	0	0	0
tcp_flag_fin	0	0	0	0	0
tcp_flags	NONE	NONE	NONE	NONE	PSH
tcp_window_size	502	64	64	502	502
tcp_seq	6.5 × 10^8^	1.4 × 10^8^	1.4 × 10^8^	6.5 × 10^8^	6.5 × 10^8^
tcp_ack	1.45 × 10^8^	6.5 × 10^8^	6.5 × 10^8^	1.4 × 10^8^	1.4 × 10^8^
tcp_payload	0	0	0	0	58
flow_relative_time	0	0	4.4 × 10^−5^	5.04	65.6
flow_inter_packet_delay	0	0	4.4 × 10^−5^	1.6 × 10^−5^	4.9
flow_packet_count	1	1	2	3	23
flow_byte_count	66	66	132	198	1902
flow_mean_packet_size	66	66	66	66	82.7
flow_packet_sizestddev	0	0	0	0	25.5
flow_packet_size_skewness	0	0	0	0	0.9
flow_packet_size_kurtosis	0	0	−3	−3	−1.1
flow_payload_size_per_packet	52	52	52	52	68.7
Label	1	1	1	1	1
Type	Attack1	Attack1	Attack1	Attack1	Attack1

The feature value *tcp_flags* is categorical to include the less frequently used flag values that are a part of the network packets. This feature value represents a concatenated value based on whether the PUSH and URGENT (URG) flags of the packet is set and NONE if both the flags are not set.

**Fig. 1:**
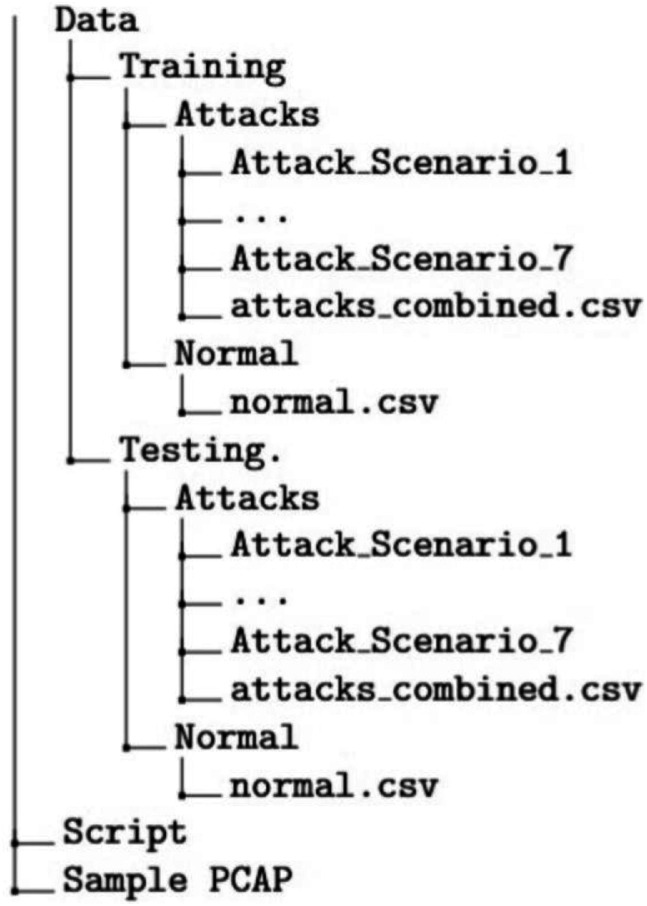
Overall directory structure.

**Fig. 2:**
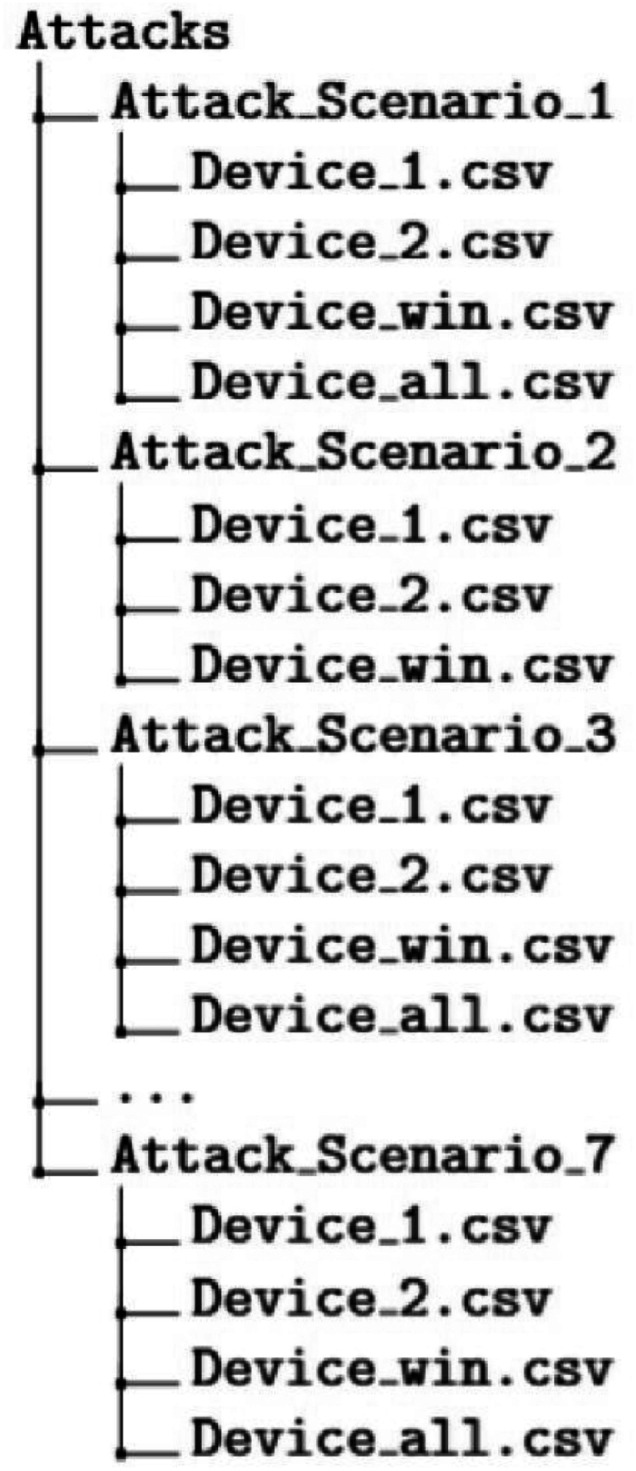
Structure of the attack subdirectory-common to training and testing directories.

The overall directory structure of the dataset is as shown in Figs. 1 and 2. The *Training* and *Testing* directories contain data from the various attack scenarios used in the experiment. Each attack scenario subdirectory contains captured data from multiple targets as shown in Fig. 2. All the attack scenario folders have data files named as 'Device_*n*.csv' with *n* having values *1,2* or *win* denoting the three targets in the network. The attack scenario directories except attack scenario 2 have a data file named as 'Device_all.csv' which stores the network flow when the particular attack scenario was launched simultaneously on all the targets. The *Attacks* directory also has a data file which combines samples from the all the attack scenarios and could be used for any classification or regression tasks. The directory named *Script* contains the code which we have used to extract the network flow data into the tabular format. A subset of filtered PCAP files which were used for training purposes are included in the folder labeled as *Sample PCAP* which could be used for user validation purposes. The included PCAP files are filtered to prevent leakage of personal data from the ubiquitous devices in the smart home network.

Each data file in the directory has two additional columns to indicate the category of the sample. The *Label* column represents the binary classification of the features with '1′ representing an attack and '0′ indicating normal feature vector. The *Type* column represents the attack scenario and the values are of the order ‘Attack*X*’ where *‘X’* represent the scenario from one to seven.

## 4 Experimental Design, Materials and Methods

The dataset was collected from a network with devices which are prevalent in a home network. The devices were chosen to impart heterogenous nature to the network with respect to the computational capability and usage patterns. The device selection for the testbed was to ensure heterogeneity and cross-domain applicability. The heterogeneous nature of the devices considered, reflects a realistic network environment commonly observed in modern smart homes and small edge-enabled enterprises. The attack types were selected to include the adversarial tactics as defined in the MITRE ATT&CK framework to emulate various attack patterns in a smart home environment. All the devices listed in Table 5 were connected to an internet connected Wi-Fi network, as in most home networks. Security of the devices was limited to the default features available in the nodes. One of the devices in the network was used as the attacker and nodes of various usage nature were selected as targets. Attacks were performed in multiple stages where initially the targets were attacked individually and finally all targets were simultaneously attacked. The network flow data in each stage was captured and processed as the dataset.

**Table 5:** Specification of the devices used in the experiment.

Sl.No	Item	OS	Specification	Quantity	Remarks
1	Laptop	Linux	Intel i7	1	Data Logger
2	Laptop	Ubuntu 22.04 LTS	Intel i5	1	Caldera server
3	Laptop	Windows 11	Intel i5	1	Target
4	Gaming Device	Raspbian	ARM	1	Target
5	Media Centre	Raspbian	ARM	1	Target
6	Smart Watch	Watch OS	Apple Watch	1	End Device
7	Smart Camera	*	imou	1	sEnd Device
8	Smart phone	iOS	iPhone	1	End Device
9	Smart plug	*	Zebronics	1	End Device
10	4 G WiFi Router	*	CP-Plus	1	End Device

### 4.1 MITRE Caldera

The attacks were performed using Caldera [11] adversary emulation platform from a node within the network. Caldera is an open source tool designed to perform adversary emulation built on the MITRE ATT&CK [12] framework. The tool was used to perform multiple attack scenarios on the network confirming to known real world attacks. The configuration of the attack scenarios is based on the real-world tactics, techniques and procedures (TTP) which provides standardization of the attacks. The attack scenarios once configured can be performed with minimal intervention thereby ensuring uniformity of the procedures across the devices.

### 4.2 Experiment setup

**Fig. 3:**
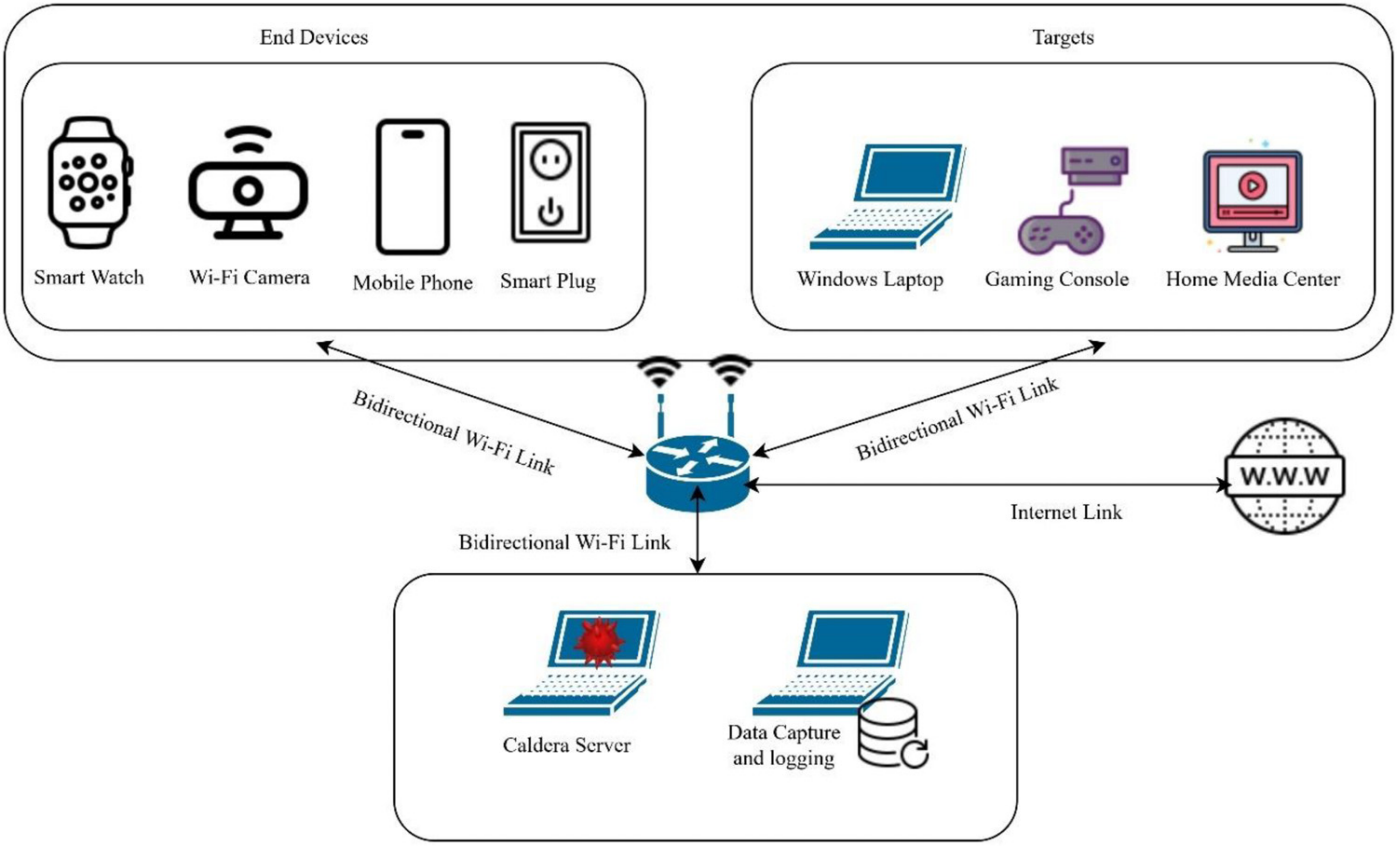
Schematic representation of smart home network testbed.

The smart home network used for the experiment used a range of devices which were chosen to replicate the devices found in a typical smart home. The devices were configured with necessary software to enable typical usages in a household, as listed in Table 5. The attacker is presumed to have gained initial access to one of the systems which is being used to launch further attacks within the network. Among all the devices present in the network given in Table 5, some are used as targets by the attacker while other devices are actively contributing to the overall dynamics of the network. The network consisted of a smart plug, a mobile phone, a Wi-Fi camera, a smart watch, a laptop, a gaming device installed with Retro pi [13] to emulate a gaming platform connected to the network and a media center device installed with Kodi [14] to allow for activities such as video streaming. A schematic representation of the network is given in Fig. 3.

All the devices were connected to the network irrespective of the target attack node and the attack pattern thereby providing a realistic flow of data in the network. The Caldera server was installed on one of the machines in the network and was marked as the attacker system. The adversary emulation performed each attack scenario individually on each target followed by simultaneous attack on all the targets. This was repeated for each of the attack scenarios.

**Fig. 4:**
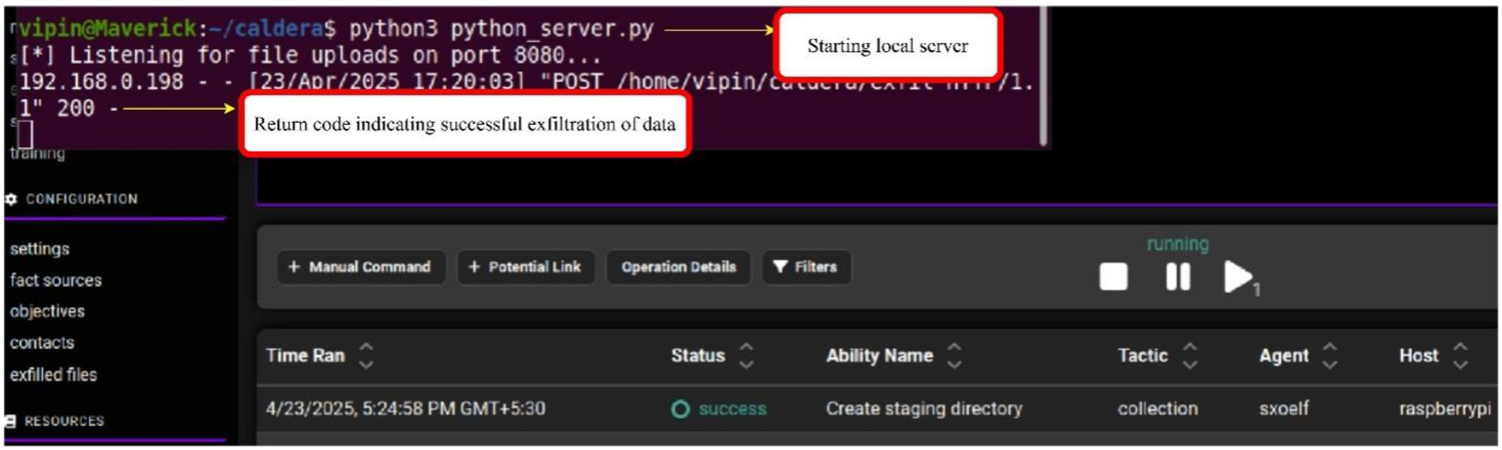
Terminal output from Caldera attacker for attack scenario seven.

The testing set of each attack scenario was developed independent of the experiments for capturing the training samples to avoid data leakages to the training set. The normal traffic capture for testing set was also captured using the same methodology of the training set but with a shorter capture duration. A screen grab of the caldera machine terminal during the attack scenario seven is shown in Fig. 4. The attack was able to offload a sensitive file to a local server to emulate the exfiltration process in the local network.

### 4.3 Attack scenarios

**Fig. 5:**
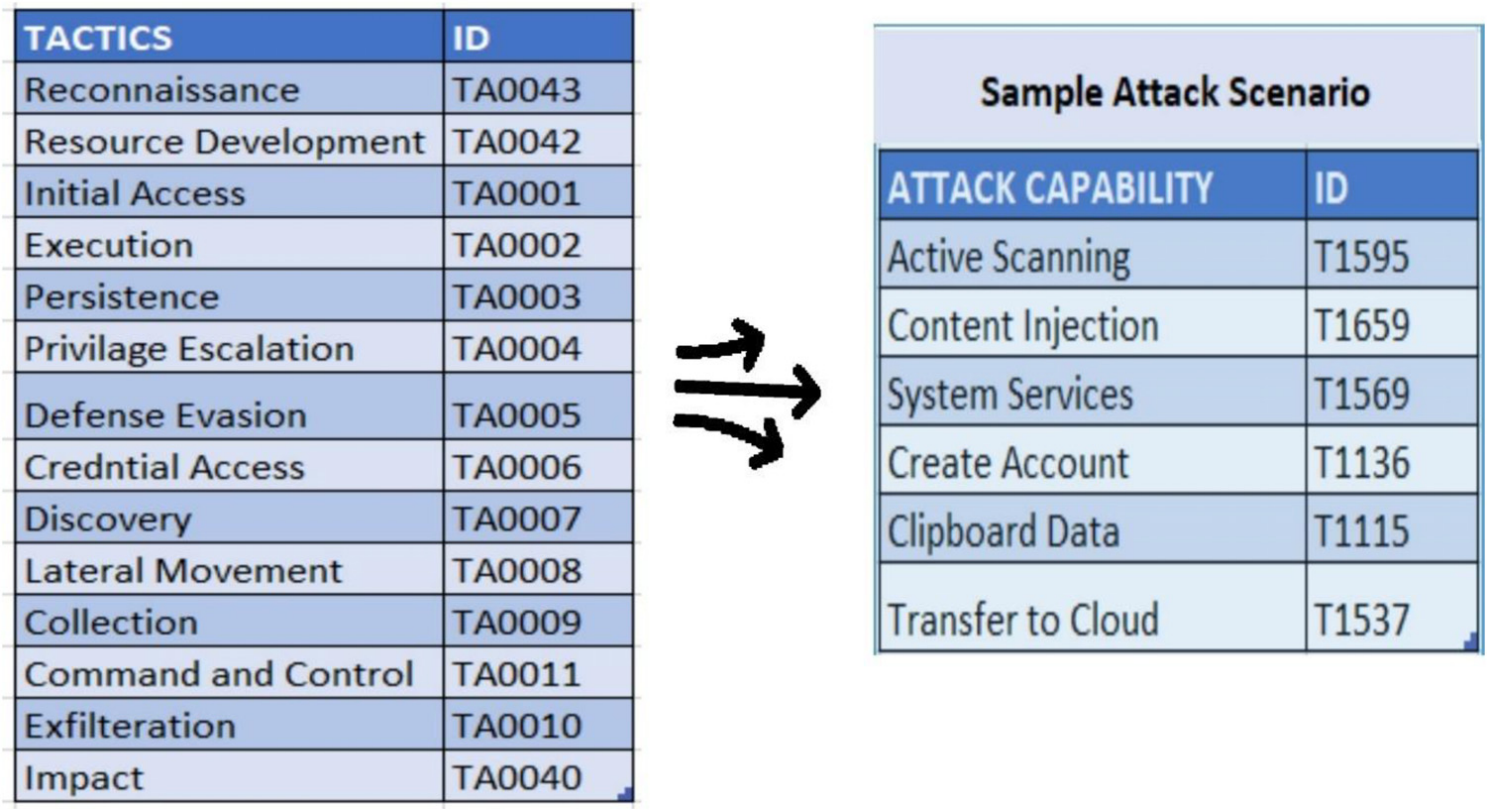
Creating attack scenarios by selecting attack capabilities from various attack tactics.

Attack scenarios define the capability of an adversary in the network. Each scenario contains multiple attack capabilities which are defined in the standard MITRE ATT&CK tactics as depicted in Fig. 5. Each of the attacks reflect the tactics, techniques and procedures used by real world adversaries. The attack scenarios created in the experiment are made to infiltrate the targets and perform multiple stages of attack in each scenario. Each attack scenario has a different expected outcome but the attacks could be repeated based on whether it is one of the stages in the attack scenario. The network data that was extracted from such an environment is novel as it captures the network characteristics when multiple stages of attacks are performed on the devices.

#### 4.3.1 Attack scenario one

The attack capabilities in the scenario executed the general capabilities such as clearing various logs, disabling system firewall, creating hidden directories, and editing the timestamps of certain directories. The sequence of attacks in scenario one ensured that the traces of any malicious activity is either removed or hidden from plain view. The attacks ensured that hidden directories are created on the target machine and pushed malicious executables. The attack is labeled as class one in the dataset. The feature statistics for this scenario is given in Table 6.

**Table 6:** Feature statistics for numerical features of attack scenario one.

Feature	Training			Testing		
	Min	Max	Avg.	Min	Max	Avg.
flow_byte_count	55	≈ 30.73 × 10^3^	≈ 10.23 × 10^3^	55	≈ 15.07 × 10^3^	≈ 56.16 × 10^2^
flow_inter_packet_delay	0	36.09	2.478	0	23.68	2.66
flow_mean_packet_size	54.33	106.06	82.91	55	114.45	82.07
flow_packet_count	1	317	117.47	1	185	66.96
flow_packet_size_kurtosis	−3	50.12	16.02	−3	51.79	11.17
flow_packet_size_skewness	−2.04	6.82	3.5	−1.154	6.75	2.81
flow_packet_size_stddev	0	112.89	51.145	0	121.69	44.44
flow_payload_size_per_packet	40.33	92.064	68.91	41	100.45	68.071
flow_relative_time	0	663.57	275.69	0	369.24	179.7
ip_frag	0	0	0	0	0	0
ip_id	4088	62673	34116.04	24221	60656	43062.26
ip_protocol	6	6	6	6	6	6
ip_ttl	64	128	72.11	64	128	73.98
packet_length	54	588	87.91	54	588	88.66
tcp_ack	≈ 14.54 × 10^7^	≈ 40.95 × 10^8^	≈ 19.72 × 10^8^	≈ 15.50 × 10^7^	≈ 40.65 × 10^8^	≈ 26.03 × 10^8^
tcp_flag_ack	1	1	1	1	1	1
tcp_flag_fin	0	0	0	0	0	0
tcp_flag_rst	0	0	0	0	0	0
tcp_flag_syn	0	0	0	0	0	0
tcp_payload_size	0	522	24.57	0	522	26.11
tcp_seq	≈ 14.54 × 10^7^	≈ 40.95 × 10^8^	≈ 18.79 × 10^8^	≈ 15.50 × 10^7^	≈ 40.65 × 10^8^	≈ 24.11 × 10^8^
tcp_window_size	63	502	≈ 18.79 × 10^9^	63	502	218.4

#### 4.3.2 Attack scenario two

The methods in attack scenario two tried to exfiltrate data to a cloud environment. The capabilities in the attack scenario two was limited to push files to a known GitHub repository. The attacks archived sensitive files which were identified in another attack and push those files to the outside network. The attacks were able to transfer two files of varying sizes to a repository. The feature statistics is given in Table 7.

**Table 7:** Feature statistics for numerical features of attack scenario two.

Feature	Training			Testing		
	Min	Max	Avg.	Min	Max	Avg.
flow_byte_count	55	≈ 13.04 × 10^6^	≈ 42.79 × 10^5^	66	≈ 56.86 × 10^4^	≈ 11.27 × 10^4^
flow_inter_packet_delay	0	30.06	0.01	0	60.76	0.53
flow_mean_packet_size	54.4	1424	973.035	66	1424	683.26
flow_packet_count	1	9171	3695.58	1	501	124.45
flow_packet_size_kurtosis	−3	998.79	439.59	−3	16.55	2.45
flow_packet_size_skewness	−31.23	28.31	−7.73	−2.43	4.25	0.42
flow_packet_size_stddev	0	679	59.88	0	677.01	399.67
flow_payload_size_per_packet	40.41	1410	959.04	52	1410	669.26
flow_relative_time	0	173.57	24.93	0	91.95	22.17
ip_frag	0	0	0	0	0	0
ip_id	0	65535	24323.28	0	65465	19918.91
ip_protocol	6	6	6	6	6	6
ip_ttl	49	128	61.77	48	64	55.96
packet_length	54	1424	979.11	54	1424	678.07
tcp_ack	0	≈ 38.37 × 10^8^	≈ 25.26 × 10^8^	0	≈ 40.28 × 10^8^	≈ 20.38 × 10^8^
tcp_flag_ack	0	1	0.99	0	1	0.98
tcp_flag_fin	0	1	0	0	1	0.01
tcp_flag_rst	0	1	0	0	1	0.01
tcp_flag_syn	0	1	0	0	1	0.01
tcp_payload_size	0	1370	916.11	0	1358	611.94
tcp_seq	≈ 50.78 × 10^7^	≈ 38.37 × 10^8^	≈ 20.84 × 10^8^	≈ 49.61 × 10^6^	≈ 17.81 × 10^8^	≈ 40.28 × 10^8^
tcp_window_size	0	65535	3988.49	0	65535	1017.84

#### 4.3.3 Attack scenario three

Attack scenario three used strategies by which attackers gain insights about the network and the connected nodes. The attacks included steps such as collecting the Address Resolution Protocol Information, scanning Wi-Fi networks, and removing activity log. This information is crucial in later stages where specific details about the device can be utilized to plan the attack tactics accordingly. The attacks were designed with network profiling and tried to identify previously connected networks, other devices in the network along with the credentials for network access. The attack scenario mechanisms could also be used to ascertain whether such probing activities would trigger attack notifications in the network. The attack scenario draws importance from the fact that home networks can contain devices with known default settings which could be used to attack the network. The attack scenario feature statistics is given in Table 8. The samples for this scenario are represented using label three in the dataset.

**Table 8:** Feature statistics for numerical features of attack scenario three.

Feature	Training			Testing		
	Min	Max	Avg.	Min	Max	Avg.
flow_byte_count	55	≈ 10.37 × 10^4^	≈ 23.61 × 10^3^	55	≈ 57.41 × 10^3^	≈ 12.77 × 10^3^
flow_inter_packet_delay	0	21.06	2.35	0	27.06	2.15
flow_mean_packet_size	54.33	774.58	214.7	54.5	785.93	191.45
flow_packet_count	1	245	99.3	1	147	52.35
flow_packet_size_kurtosis	−3	49.29	5.46	−3	31.45	2.77
flow_packet_size_skewness	−1.5	7.04	2.13	−1.5	5.56	1.37
flow_packet_size_stddev	0	708.46	239.19	0	721.03	155.25
flow_payload_size_per_packet	40.33	760.58	200.7	40.5	771.94	177.45
flow_relative_time	0	538.79	240.6	0	223.6	91.71
ip_frag	0	0	0	0	0	0
ip_id	2613	62418	30394.98	24340	60901	45574.68
ip_protocol	6	6	6	6	6	6
ip_ttl	64	128	70.69	64	128	81.97
packet_length	54	1514	254.44	54	1514	231.74
tcp_ack	≈ 25.38 × 10^6^	≈ 40.95 × 10^8^	≈ 23.75 × 10^8^	≈ 15.50 × 10^7^	≈ 36.41 × 10^8^	≈ 22.07 × 10^8^
tcp_flag_ack	1	1	1	1	1	1
tcp_flag_fin	0	0	0	0	0	0
tcp_flag_rst	0	1	0	0	0	0
tcp_flag_syn	0	0	0	0	0	0
tcp_payload_size	0	1460	190.74	0	1448	170.8
tcp_seq	≈ 25.38 × 10^6^	≈ 40.95 × 10^8^	≈ 23.06 × 10^8^	≈ 15.50 × 10^7^	≈ 36.41 × 10^8^	≈ 22.55 × 10^8^
tcp_window_size	0	502	251	49	502	229.61

#### 4.3.4 Attack scenario four

The attack tactics defined in the scenario four targets the system to retrieve core system based specification. The attack capabilities in the scenario used techniques to collect the details about the environment variables, locating specific folders, discovering the particulars of the Operating System, and discovering various permissions in the system. The attack steps were chosen to gather critical host intelligence on multiple types of Operating systems. The presence of outdated system software in a home network can be identified by the attacker which would help to craft other set of malicious attempts focused on the gained knowledge. The attack scenario also tried to list basic permissions available for the users and identify the environment variables used by the system. The feature statistics is given in Table 9.

**Table 9:** Feature statistics for numerical features of attack scenario four.

Feature	Training			Testing		
	Min	Max	Avg.	Min	Max	Avg.
flow_byte_count	55	≈ 50.43 × 10^4^	≈ 91.95 × 10^3^	55	≈ 18.74 × 10^4^	≈ 45.89 × 10^3^
flow_inter_packet_delay	0	41.48	2.92	0	45.4	3.04
flow_mean_packet_size	54.5	1335.16	615.59	54.33	1391.86	521.94
flow_packet_count	1	407	104.27	1	171	56.52
flow_packet_size_kurtosis	−3	46.63	7.84	−3	45.31	6.1
flow_packet_size_skewness	−2.27	6.8	1.16	−2.96	6.67	1.25
flow_packet_size_stddev	0	709.37	317.18	0	715.9	316.63
flow_payload_size_per_packet	40.5	1321.16	601.59	40.33	1377.85	507.94
flow_relative_time	0	608.19	186.2	0	343.42	134
ip_frag	0	0	0	0	0	0
ip_id	3027	63140	34263.76	2765	63254	43018.56
ip_protocol	6	6	6	6	6	6
ip_ttl	64	128	69.66	64	128	68.93
packet_length	54	1514	602.72	54	1514	586.81
tcp_ack	≈ 32.74 × 10^7^	≈ 42.92 × 10^8^	≈ 24.84 × 10^8^	≈ 39.55 × 10^7^	≈ 30.89 × 10^8^	≈ 17.25 × 10^8^
tcp_flag_ack	1	1	1	1	1	1
tcp_flag_fin	0	0	0	0	0	0
tcp_flag_rst	0	0	0	0	0	0
tcp_flag_syn	0	0	0	0	0	0
tcp_payload_size	0	1448	538.41	0	1448	522.31
tcp_seq	≈ 32.74 × 10^7^	≈ 42.92 × 10^8^	≈ 25.14 × 10^8^	≈ 39.55 × 10^7^	≈ 30.89 × 10^8^	≈ 17.46 × 10^8^
tcp_window_size	0	502	317.4	0	502	311

#### 4.3.5 Attack scenario five

A set of sophisticated set of attacks ranging from initial reconnaissance, staging of stolen information to launching of apps were performed as a part of the attack scenario five. The attacks progressed through various stages like identifying specific files, discovering antivirus applications, finding sensitive files, and trying to exfiltrate the data to a server. A local server was setup in the network to capture such data exfiltration attempts. The attacks exploit the known vulnerabilities in the systems to gain access to the sensitive data. The attacks also included privilege escalation techniques using which the entire node can be controlled. The lateral movement capability of the attacks coupled with staging capability of files in a home environment could provide opportunity for an attacker to steal information from connected smart devices in the network. The feature statistics for this scenario are given in Table 10. The samples for this attack scenario are labeled as class five in the dataset.

**Table 10:** Feature statistics for numerical features of attack scenario five.

Feature	Training			Testing		
	Min	Max	Avg.	Min	Max	Avg.
flow_byte_count	55	≈ 76.16 × 10^3^	≈ 17.25 × 10^3^	54	≈ 24.58 × 10^4^	≈ 42.75 × 10^3^
flow_inter_packet_delay	0	60.59	4.15	0	23.97	2.56
flow_mean_packet_size	54.3	436.47	126.46	54	642.74	155.65
flow_packet_count	1	369	132.97	1	922	271.72
flow_packet_size_kurtosis	−3	56.86	10.92	−3 66	43	8.78
flow_packet_size_skewness	−1.15	6.75	2.93	−1.5	7.52	2.72
flow_packet_size_stddev	0	611.68	131.35	0	667.34	183.3
flow_payload_size_per_packet	40.3	422.47	112.46	40	628.74	141.65
flow_relative_time	0	1271.18	488.38	0	1361.18	508.88
ip_frag	0	0	0	0	0	0
ip_id	3187	63419	34529.49	25837	63519	45785.27
ip_protocol	6	6	6	6	6	6
ip_ttl	64	128	71.95	64	128	70.42
packet_length	54	1514	138.63	54	1514	158.98
tcp_ack	≈ 32.77 × 10^7^	≈ 42.92 × 10^8^	≈ 27.13 × 10^8^	≈ 39.55 × 10^7^	≈ 30.89 × 10^8^	≈ 13.91 × 10^8^
tcp_flag_ack	1	1	1	1	1	1
tcp_flag_fin	0	0	0	0	0	0
tcp_flag_rst	0	0	0	0	0	0
tcp_flag_syn	0	0	0	0	0	0
tcp_payload_size	0	1460	75.12	0	1460	94.88
tcp_seq	≈ 32.77 × 10^7^	≈ 42.92 × 10^8^	≈ 26.66 × 10^8^	≈ 39.55 × 10^7^	≈ 30.89 × 10^8^	≈ 13.92 × 10^8^
tcp_window_size	53	501	320.18	53	502	269.56

#### 4.3.6 Attack scenario six

The feature statistics described in Table 11 refers to a set of attacks with primary focus of active user monitoring after making the initial access. The attack vectors had the capability to take screenshot of the system, access clipboards, and web bookmarks on selected software environments. The data capture mechanisms depended on legitimate API calls rather than illicit means to extract the information. The constituent attack stages like scanning Wi-Fi details and extracting data to another system was also a part of other attack scenarios.

**Table 11:** Feature statistics for numerical features of attack scenario six.

Feature	Training			Testing		
	Min	Max	Avg.	Min	Max	Avg.
flow_byte_count	55	≈ 26.63 × 10^3^	≈ 98.38 × 10^2^	55	≈ 17.25 × 10^4^	≈ 11.97 × 10^3^
flow_inter_packet_delay	0	21.61	2.96	0	35.62	3.31
flow_mean_packet_size	54.5	230.43	83.04	54.2	1460.88	222.72
flow_packet_count	1	298	118.25	1	131	49.66
flow_packet_size_kurtosis	−3	43.92	13.26	−3	45.32	9.61
flow_packet_size_skewness	−1.155	6.4	3.34	−4.84	6.85	2.26
flow_packet_size_stddev	0	271.44	51.58	0	720	96.31
flow_payload_size_per_packet	40.5	216.43	69.04	40.2	1446.88	208.72
flow_relative_time	0	805.47	352.49	0	470.15	152.69
ip_frag	0	0	0	0	0	0
ip_id	10440	65004	36419.65	0	64207	35449.78
ip_protocol	6	6	6	6	6	6
ip_ttl	64	128	70.12	64	128	71.47
packet_length	54	910	83.82	54	1514	231.9
tcp_ack	≈ 56.98 × 10^6^	≈ 38.31 × 10^8^	≈ 20.68 × 10^8^	0	≈ 40.85 × 10^8^	≈ 22.33 × 10^8^
tcp_flag_ack	1	1	1	0	1	0.99
tcp_flag_fin	0	0	0	0	1	0
tcp_flag_rst	0	1	0	0	1	0
tcp_flag_syn	0	0	0	0	1	0
tcp_payload_size	0	856	20.03	0	1448	168.88
tcp_seq	≈ 56.98 × 10^6^	≈ 38.31 × 10^8^	≈ 21.58 × 10^8^	≈ 37.28 × 10^7^	≈ 40.85 × 10^8^	≈ 23.76 × 10^8^
tcp_window_size	0	502	237.04	0	65160	475.19

#### 4.3.7 Attack scenario seven

The attacks included in the attack scenario seven was crafted to search for sensitive files staged during earlier attack attempts and exfiltrating the same to a server. As a part of the experiment a local server was setup on the attacker machine so as to gather the information from the attack. The feature statistics are described in Table 12.

**Table 12:** Feature statistics for numerical features of attack scenario seven.

Feature	Training			Testing		
	Min	Max	Avg.	Min	Max	Avg.
flow_byte_count	54	≈ 99.12 × 10^2^	≈ 42.41 × 10^2^	54	≈ 19.55 × 10^4^	≈ 13.85 × 10^3^
flow_inter_packet_delay	0	71.69	4.39	0	120.82	4.15
flow_mean_packet_size	54	124.13	80.7	54	1447.58	214.05
flow_packet_count	1	122	51.68	1	137	46.14
flow_packet_size_kurtosis	−3	50.13	8.32	−3	53.46	7.09
flow_packet_size_skewness	−1.15	6.75	2.66	−4.24	7.19	1.99
flow_packet_size_stddev	0	191.07	42.25	0	694.71	83.8
flow_payload_size_per_packet	40	110.13	66.7	40	1433.58	200.05
flow_relative_time	0	578.86	201.61	0	480.15	144.79
ip_frag	0	0	0	0	0	0
ip_id	14046	64405	39731.65	0	54912	28850.06
ip_protocol	6	6	6	6	6	6
ip_ttl	64	128	69.66	64	128	71.23
packet_length	54	919	82.69	54	1514	227.35
tcp_ack	≈ 35.96 × 10^6^	≈ 41.81 × 10^8^	≈ 19.60 × 10^8^	0	≈ 34.44 × 10^8^	≈ 17.30 × 10^8^
tcp_flag_ack	1	1	1	0	1	1
tcp_flag_fin	0	0	0	0	1	0
tcp_flag_rst	0	1	0	0	0	0
tcp_flag_syn	0	0	0	0	1	0
tcp_payload_size	0	865	18.66	0	1448	163.8
tcp_seq	≈ 35.96 × 10^6^	≈ 41.81 × 10^8^	≈ 19.51 × 10^8^	≈ 14.60 × 10^7^	≈ 34.44 × 10^8^	≈ 15.14 × 10^8^
tcp_window_size	63	502	251.69	59	65160	467.55

#### 4.3.8 Normal traffic

The normal traffic in the experiment was captured from the network interactions made by the devices in the network and from the browsing traffic from the computing nodes. This capturing was planned to identify the normal traffic within the network along with the traffic to the internet as the attack scenarios had local traffic and used internet traffic for some attacks. The normal traffic feature statistics from the network are given in Table 13.

**Table 13:** Feature statistics for numerical features of normal traffic.

Feature	Training			Testing		
	Min	Max	Avg.	Min	Max	Avg.
flow_byte_count	54	≈ 93.27 × 105	≈ 11.33 × 105	54	≈ 31.77 × 105	≈ 45.25 × 104
flow_inter_packet_delay	0	600.45	2.01	0	58.47	0.17
flow_mean_packet_size	54	1424	744.64	54	1404	837.41
flow_packet_count	1	6574	901.76	1	2279	381.31
flow_packet_size_kurtosis	−3	671.98	47.4	−3	333.81	40.66
flow_packet_size_skewness	−17.27	25.23	−1.32	−14.17	18.32	−1.02
flow_packet_size_stddev	0	685	261.16	0	672.01	234.07
flow_payload_size_per_packet	40	1410	730.64	40	1390	823.41
flow_relative_time	0	2200.27	99.98	0	89.58	3.99
ip_frag	0	0	0	0	0	0
ip_id	0	65535	30072.95	0	65356	11176.9
ip_protocol	6	6	6	6	6	6
ip_ttl	38	246	89.13	41	245	117.76
packet_length	54	1424	735.96	54	1424	869.92
tcp_ack	0	≈ 42.95 × 108	≈ 22.51 × 108	0	≈ 42.80 × 108	≈ 21.79 × 108
tcp_flag_ack	0	1	0.98	0	1	0.99
tcp_flag_fin	0	1	0.02	0	1	0
tcp_flag_rst	0	1	0.01	0	1	0
tcp_flag_syn	0	1	0.03	0	1	0.02
tcp_payload_size	0	1370	670.24	0	1358	809.28
tcp_seq	≈ 34.32 × 105	≈ 42.95 × 108	≈ 21.45 × 108	≈ 14.38 × 106	≈ 42.80 × 108	≈ 19.41 × 108
tcp_window_size	0	65535	2451.66	0	65535	1475.21

### 4.4 Machine learning based attack detection workflow

**Fig. 6:**
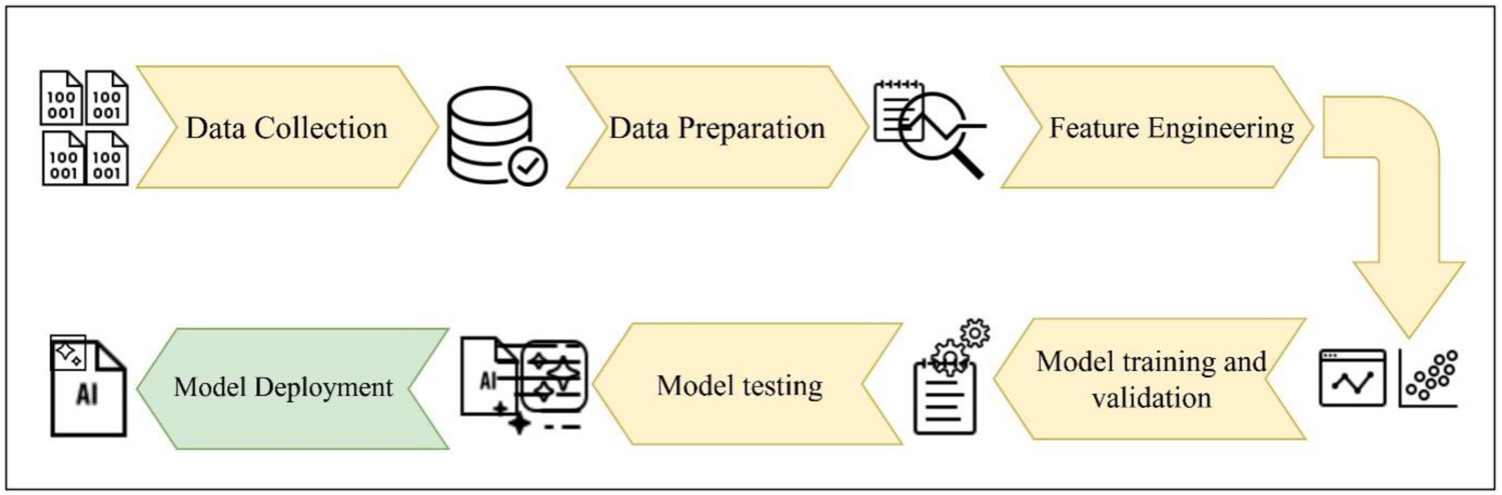
Machine learning workflow for building attack detection systems.

The dataset proposed in this paper can be utilized to develop machine learning models which can detect the presence of the attack samples and ensure protection for the data and devices in the home network. Typical machine learning based system design workflow to detect and classify attacks from network flow information from other type of networks is given in Fig. 6. This workflow can easily be adopted in the case of a home network also. Once the entire data is prepared, the features can be engineered so as to reduce redundancy and improve the efficiency of the model. Model training and validation could be thought of as an iterative step coupled to the feature engineering stage as the validation results can be used to select the best features and samples in the data. The original data also be converted to other forms which can suit the selected algorithms. The work presented in [15] had converted the original data into an image form so as to fit the input requirements of their proposed model. The next stage involves deploying the model to an environment so that the model can start classifying the new data. The option of retraining the model based on novel data may also be explored to make the system robust.The researchers can also explore the option of optimizing the machine learning model so that a compressed model can be deployed on low powered devices in the home network. Although the dataset has been generated for application in smart home security research, its applicability extends beyond a typical smart home environment. The experimental test bed incorporates heterogeneous end devices, reflecting characteristics common in edge-enabled enterprise networks. In addition, the adversarial scenarios considered in this study were implemented based on the MITRE ATT&CK Enterprise Tactics, which emulate the attacks observed in real-world enterprise intrusions. Potential use cases of the proposed dataset include its utility for benchmarking both classical and learning-based intrusion detection systems subject to multi-stage attacks. The dataset proposed in this study also provides flow-level features extracted from PCAP files making it transferable to broader intrusion detection research contexts.

A random forest [16]based attack detection and classification system has been designed to provide base line results for researchers interested in building more advanced models. The reported metrics in Table 15 correspond to a weighted evaluation scheme to address variations in the class counts in the dataset. The hyperparameters reported in Table 14 were arrived at using grid search.

**Table 14:** Model hyperparameters.

Parameters	Binary Class	Multi-class
n_estimators	200	200
min_samples_split	10	3
min_samples_leaf	3	1
max_features	log2	all
max_depth	22	22

The performance of the system is shown in Table 15. Five performance measures namely precision, recall, accuracy, specificity and F1 score were considered.

**Table 15:** Classification metrics by using a random forest algorithm.

	Binary	Multiclass
**Precision**	0.8769	0.8201
**Recall**	0.9840	0.7749
**Accuracy**	0.9345	0.7749
**Specificity**	0.8980	0.9588
**F1**	0.9274	0.7849

## Limitations

The experimental testbed and the attack scenarios were crafted to model a conventional Smart home network with a set of representative smart home devices and a set of multiple attacks which expose the data and systems to an unauthorized person. The dataset does not capture the host level interactions on the systems under attack during the attack scenarios which may enhance the capability of a detection system to classify the attack scenario. The data samples captured for each attack scenario in the data set is not of identical size as the data exchanged during each scenario was different when compared with other attack scenarios. The user behaviour pattern on the devices were not analysed during the experiments.

## Ethics Statement

The authors have read and understood the ethical requirements for publication in Data in Brief and confirm that the current work does not involve human subjects, animal experiments, or any data collected from social media platforms.

## Credit Author Statement

**Vipin Das**: Data curation, Software, Visualization, Writing- original draft preparation. **Binoy B Nair**: Conceptualization, Methodology, Reviewing and Editing.

## Declaration of Competing Interest

The authors declare that they have no known competing financial interests or personal relationships that could have appeared to influence the work reported in this paper.

## Data Availability

(Mendeley Data)Smart Home Intrusion Detection Dataset (Original data).
